# Food-dependent activation of one-carbon metabolism during heat shock in *Mytilus californianus*

**DOI:** 10.1242/jeb.252105

**Published:** 2026-08-11

**Authors:** R. Frank Fabela, Melissa A. May, Lars Tomanek

**Affiliations:** ^1^ California Polytechnic State University, Department of Biological Sciences, 1 Grand Ave., San Luis Obispo, CA 93407, USA; ^2^ Florida Gulf Coast University, Department of Marine and Earth Sciences, 10501 FGCU Blvd. S, Fort Myers, FL 33965, USA

**Keywords:** Carbohydrate metabolism, Cellular stress response, Histone modifications, *Mytilus*, One-carbon metabolism, Oxidative stress, Proteomics

## Abstract

Of the ecological factors affecting the cellular stress response, food ration has received little attention despite a likely role in stress tolerance limits. To test the interactive effects of food and temperature on the proteomic response to heat shock, the intertidal mussel *Mytilus californianus* was acclimated to four combinations of nearshore (low) and aquaculture (high) phytoplankton levels, combined with low (20°C) and high (30°C) aerial temperatures during daytime low tides. Whole mussels were then exposed to an acute (6 h), aerial heat stress (33°C) and allowed to recover for 1 and 25 h in pre-exposure conditions. Proteomic changes in the gill before and after heat shock were measured using liquid chromatography–mass spectrometry and label-free quantification. Mussels acclimated to low temperature–low food elicited a greater heat shock response compared with other acclimation groups, increasing protein levels of core carbohydrate and sulfide metabolism. These glycolytic metabolites proposedly feed into one-carbon metabolism (e.g. folate and methionine cycles) and connecting pathways, which also increased abundance and likely contribute to purine synthesis, methylation and transsulfuration. These mussels additionally increased levels of proteins scavenging hydrogen peroxide, chelating ferrous iron and maintaining reduced glutathione levels. Proteins modifying chromatin (histone) structure and regulating transcription likely link the metabolic changes to chromatin modifications, specifically through S-adenosylmethionine-dependent methyltransferases, which may contribute to repairing DNA damage caused by oxidative stress. Overall, our results suggest a food-dependent link between metabolism and epigenetic modifications that we hypothesize may function as an autoregulatory feedback mechanism during the cellular stress response under tidally fluctuating conditions.

## Teaser

**Summary:** A food-dependent link between one-carbon metabolism and epigenetic modifications may function as an autoregulatory feedback mechanism to repair DNA damage and scavenge oxidants during heat stress.

## INTRODUCTION

The cellular stress response (CSR) elucidated the mechanisms utilized during an acute temperature increase and initiated many of the early discoveries of the molecular biology era (Lindquist, 1986; Ritossa, 1962). Since then, the CSR protein complement has been extended to include a number of functional categories beyond protein folding chaperones, such as other proteostasis proteins involved in protein degradation, metabolic pathways producing ATP and metabolites, controlling redox balance and DNA repair (Kültz, 2005; Somero et al., 2017). Early comparative studies investigated the importance of the CSR for organisms in their native habitat and thermal range. In these, patterns identifying the onset of the response correlated with seasonal acclimatization and acclimatory plasticity (Feder and Hofmann, 1999; Tomanek and Somero, 1999) provided insight into how the CSR could help organisms to adjust to climate change (Tomanek, 2010). Although these studies have advanced our understanding of the CSR, the role of food ration by itself or in combination with temperature acclimation has been largely neglected, although the importance of food level and diet composition on ectotherm thermal performance in general has been documented (Hardison and Eliason, 2024). Here, we address this void by evaluating the proteomic response of intertidal mussels that have been acclimated to low or high food and thermal regimes to investigate how marine organisms cope with a highly fluctuating environment.

The marine mussel, *Mytilus* spp., is an abundant member of the rocky intertidal zone and acts as a ‘foundation species’ that creates dense scaffolding, providing microhabitats and protection for other organisms, effectively increasing biodiversity and species richness (Lafferty and Suchanek, 2016). Extensively studied in their ecology, anatomy and physiology, *Mytilus* have been used as models in environmental stress responses and recovery studies for decades (Bayne, 2017; Gosling, 1992). Studies on RNA/DNA ratios, enzyme activities and carotenoids between different intertidal and geographical locations varying in phytoplankton abundance or feeding time demonstrated the importance of food availability for the ecology of *Mytilus* under field conditions (Dahlhoff and Menge, 1996; Dowd et al., 2013; Lesser et al., 2010; Petes et al., 2008; Phillips, 2004). The importance of food ration at the organismal level was demonstrated by studies that showed that higher food ration can confer improved thermal stress tolerance as much as acclimation to higher temperatures in *Mytilus*, affecting survival and feeding rates under aerial heat stress (Fitzgerald-Dehoog et al., 2012; May et al., 2021; Schneider et al., 2010). Documented and predicted changes of net primary production in the North Atlantic and decreases in algal blooms in the California Current System, where *Mytilus* occur, suggest that food ration due to global change could affect the CSR (Dai et al., 2023; Moore et al., 2018).

Here, we present the proteomic response to acute heat stress, using a liquid chromatography tandem mass spectrometry (LC-MS/MS) label-free quantification approach (Michalski et al., 2011). Our results present a strong effect of food-by-temperature acclimation on maintaining energy homeostasis, redox balance, chromatin modifications and DNA repair, mediated by one-carbon metabolism. Changes in mRNA processing and selectivity, ribosomal structure, molecular chaperoning and protein degradation will be dealt with in a companion paper (Tomanek et al., 2026 preprint), as will be the effects of food-dependent sirtuins (May et al., 2026). These dual-stressor results suggest that food ration is a critical modulator of the stress response of marine organisms and that both thermal history and food ration influence the severity and cellular response to acute thermal stress.

## MATERIALS AND METHODS

### Mussel collection and experimental design

**Fig. 1.**
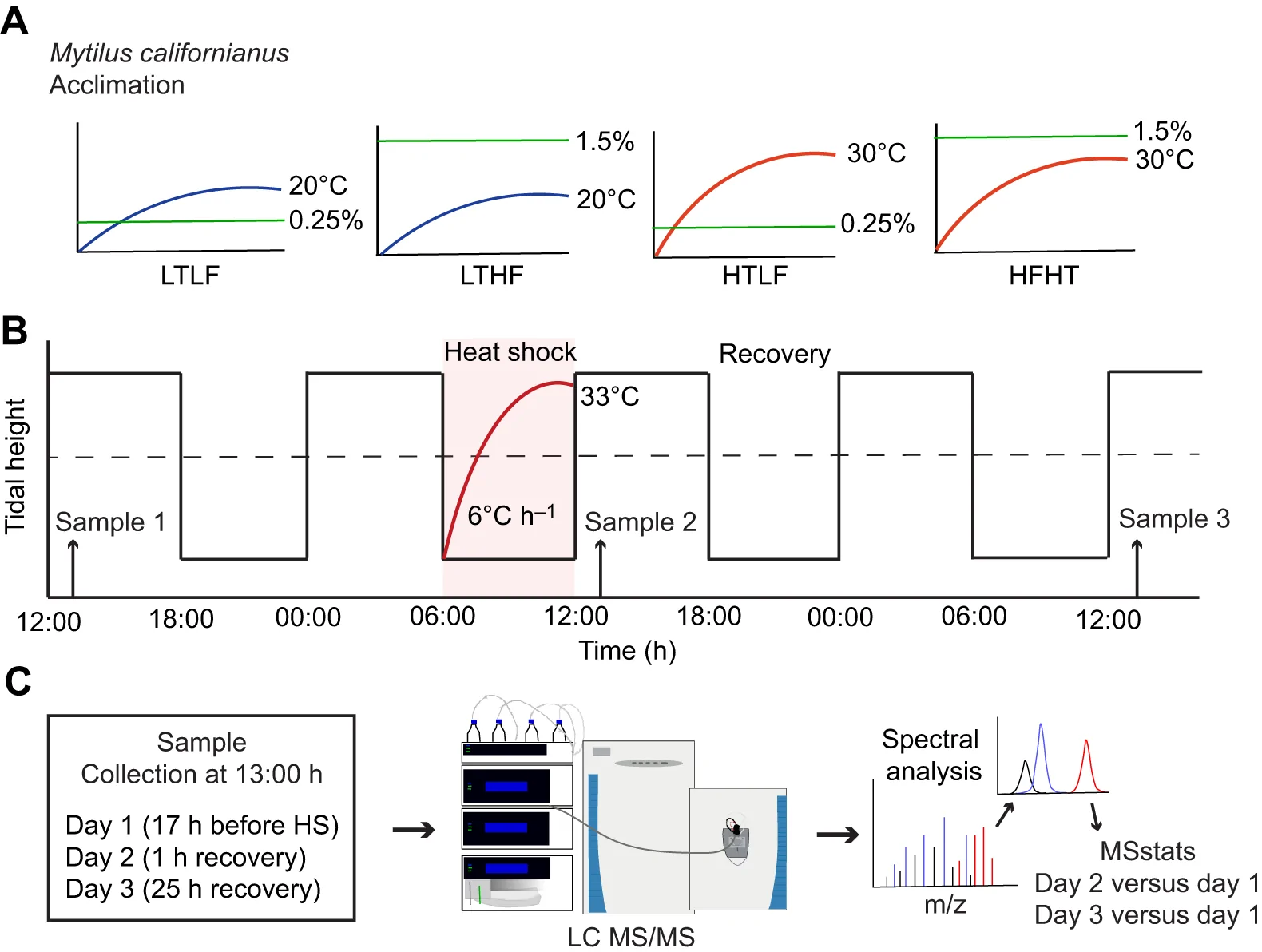
**Experimental design and sample collections for *Mytilus californianus* heat shock exposure and recovery.** (A) Acclimation conditions for all four treatments, which include combinations of low or high aerial temperature (20°C or 30°C) and low or high food ration (0.25 or 1.5% dry mass). These correspond to low temperature–low food (LTLF), low temperature–high food (LTHF), high temperature–low food (HTLF) and high temperature–high food (HTHF) groups. (B) Timing of changes in water level, sampling events and application of heat shock (33°C). (C) Proteomic workflow to analyze gill tissues using LC-MS/MS. Label-free quantification was used to determine relative protein abundance of the recovery groups compared with the baseline in MSstats (see Materials and Methods for details).

Mussel collection and experimental design were conducted as described in May et al. (2021). Briefly, adult *Mytilus californianus* Conrad 1837 (45–65 mm length) were collected from the mid-intertidal zone of Montana de Oro State Park, Los Osos, CA, USA, during low tide and acclimated in four groups at the California Polytechnic State University Center for Coastal Marine Studies (Avila, CA, USA) in flow-through, ‘tidal-simulator’ tanks that allow for control of tidal level, food ration, circadian cycles, and water and air temperatures. Mussels were acclimated for 3 weeks (Fig. 1A) to a combination of either a 20°C or 30°C aerial temperature during low tide, as well as a low (0.25% algae g^−1^ mussel dry mass) or high (1.25% algae g^−1^ mussel dry mass) ration of Shellfish Diet™ 1800 (Reed Mariculture, Campbell, CA, USA). The aerial temperature for each tank was controlled using ‘robomussels’ (Helmuth et al., 2016) to mimic the internal body temperature of the animal rather than air. A 12 h circadian clock (light: 06:00–18:00 h; dark: 18:00–06:00 h) was combined with a semi-diurnal tidal cycle [6 h low and high tide cycles each 12 h period, e.g. 06:00–12:00 h (low) and 12:00–18:00 h (high)]. Feeding occurred during high tides, with food pulsed into the tanks at 1 h increments, for a total of 10 feeding events per day. During daytime low tides, aerial temperatures were increased at a rate of 0.1°C min^−1^, while evening low tides were held at ambient temperature (18°C), a moderate heating rate under field conditions (Moyen et al., 2019). Water temperature in all tanks was 16°C. Each tank contained roughly 400 mussels, which were subsampled at each time point throughout the experiment. Following acclimation, mussels were exposed to a 33°C aerial heat shock during the daytime low tide and returned to acclimation conditions for the duration of the experiment (Fig. 1B). Gill tissue was excised from mussels (*n*=5 biological replicates) in each acclimation group at 3 h intervals throughout the experiment as part of a broader study. The gill was selected due to its roles in ventilating the mantle cavity for respiration and filter-feeding as well as its active metabolism in comparison to other tissues. Tissues were flash frozen and stored at −80°C until further analysis. Owing to the breadth of data generated, only protein abundances from mussels sampled at 13:00 h over a 3-day period – which corresponded to 17 h prior to the initiation of heat shock, and 1 and 25 h following the heat shock – were included in this paper.

### Sample preparation

Approximately 200 mg of gill tissue was homogenized in an 8 mol l^−1^ urea (Thermo Scientific, Waltham, MA, USA), 2 mol l^−1^ thiourea (Thermo Scientific), and 1% deoxycholate (Spectrum Chemicals, New Brunswick, NJ, USA) denaturing buffer to improve protein solubilization while allowing for easier removal of detergents (Feist and Hummon, 2015). Cells were lysed via sonication (3×30 s), then kept on ice for 30 min, prior to centrifugation at 17,000 ***g*** for 30 min at 4°C. Contaminants were removed via protein precipitation by adding 4× volume 10% trichloroacetic acid (Fisher Chemical, Pittsburg, PA, USA) in acetone (Fisher Chemical) and stored overnight at −20°C. Proteins were then pelleted at 17,000 ***g*** for 20 min at 4°C, washed 2× in 1 ml 100% ice-cold acetone, and resuspended in 8 mol l^−1^ urea with 150 mmol l^−1^ Tris-HCl (Fisher Chemical). Total protein concentration was determined using the Pierce™ BCA Protein Assay Kit (Thermo Scientific) according to manufacturer instructions. Aliquots containing 30 μg of total protein were reduced with dithiothreitol (4 mmol l^−1^; Thermo Scientific) and alkylated with iodoacetamide (18 mmol l^−1^; Bio-Rad, Hercules, CA, USA). Prior to digestion in solution of Pierce™ trypsin protease (Thermo Scientific), the solution was diluted to <1 mol l^−1^ urea by adding 9× volume of ammonium bicarbonate (Thermo Scientific) following the manufacturer's protocol. Finally, peptides were desalted using Pierce™ C_18_ spin columns (Thermo Scientific) according to manufacturer instructions and eluted to a final concentration of 750 ng μl^−1^ and analyzed using ultra-high-performance LC coupled with tandem MS (UHPLC-MS/MS) immediately following clean up. All samples were run in triplicate for technical replicates and 1 μl of sample was used per injection.

### UHPLC-MS/MS

Peptides were separated by reverse phase chromatography (Ultimate 3000 HPLC) on a 5 mm×300 μm C_18_ precolumn (Thermo Scientific) and then on a 150 mm×75 μm (particle size 2 μm) Acclaim PepMap 100 C_18_ LC column (Thermo Scientific). We ran a linear gradient from 1% buffer B [80% acetonitrile with 0.08% formic acid (FA); Fisher Chemical] in buffer A (0.1% FA; Fisher Chemical), to 35% buffer B in 90 min at a flow rate of 300 nl min^−1^. Eluted peptides were sprayed into the Q Exactive Plus Hybrid Quadrupole-Orbitrap MS (Thermo Scientific) using the Nanospray Flex™ Ion Source with the following parameters: spray voltage, 2.4 kV; capillary temperature, 275°C; S-lens RF level, 50. For data dependent acquisition, full MS positive ion scans were taken between 375 and 1575 *m/z* at a resolution of 70,000 with automatic gain control (AGC) target of 3×10^6^ at a maximum injection time of 100 ms. MS/MS scans were taken at a resolution of 17,500 with an AGC target of 1×10^5^ with a maximum injection time of 50 ms. The top 10 precursor ions were fragmented using collision induced fragmentation at a normalized collisional energy of 27%. Dynamic exclusion was used with a duration of 20 s, the intensity threshold was set to 1.6×10^5^, with an isolation window of 2 *m*/*z* and lock masses turned on.

### MS data processing and statistical analysis

Data were acquired with Thermo's Tune v.2.12 build 3134 and XCalibur v. 4.4.16.14 and peptides were analyzed using Proteome Discoverer v.2.3 (PD). The processing and consensus workflows were based upon those described in (Griss et al., 2019). MS Amanda was used as a database node for protein identification with the following parameters: a protein database from *Mytilus galloprovincialis* (Lamark) (NCBI taxonomy ID: 29158) was used for predicted proteins, allowing for trypsin digestion with three missed cleavages, an MS1 tolerance of 7 ppm, and an MS/MS tolerance of 0.03 Da. Settings searched for static carbamidomethyl modifications on cysteine, dynamic oxidation modifications on methionine and acetyl modifications on the N-terminus of peptides (as this study also investigated the role of NAD-dependent deacetylases). Protein ID was compared with concatenated target decoy proteins. Proteins were scored based on a false discovery rate (FDR) of *P*<0.01 for high confidence proteins and *P*<0.05 for low confidence proteins. Precursor parent ion tolerance was set to 5 ppm, with a fragment tolerance of 0.02 Da at a precision of 0.999 for high confidence peptides.

Label-free quantitation, including peptide data filtering, protein matching and statistical analysis comparisons, was performed in MSstats R package v.4.6.5 (Choi et al., 2017). Briefly, maximum peak area was used to quantify peptide abundance in PD, then unique peptides were matched to proteins using an FDR of 2% in MSstats. Only unique peptides, peptides matched to only one protein, and proteins with at least two matched peptides were used for analysis. The most intense peak area measured per peptide were log_2_ transformed and median normalized by shifting intensities by a constant to make the median constant across MS runs. Run-level summarization was performed using Tukey's median polish method, generating model estimates using peptide medians within and across technical replicates, providing robustness against outliers. Model estimation within MSstats proceeded in two steps, using a linear mixed-effects model, with both biological and technical replicates included. A whole plot that includes the overall model median, plus the condition and subject-by-condition variation and the run variation, and a subplot that includes the run variation and error term for each feature (i.e. peptide intensity) by run combination. For reasons explained in Kohler et al. (2023), the subplot can be analyzed with an ANOVA-style summarization, while the whole plot estimation is based on a restricted maximum likelihood (REML) procedure. Differential abundance (α=0.05) was determined by comparing the response of mussels 1 or 25 h after heat shock to the relative baseline acclimation conditions (Fig. 1C), within acclimation group.

The *P*-values were adjusted for multiple comparisons based on Benjamini and Hochberg (1995), which also represents the FDR. Model assumptions for normal distribution and constant variance of measurement errors can be verified visually in MSstats. Heatmaps were produced in R utilizing the ‘pheatmap’ (https://CRAN.R-project.org/package=pheatmap) and ‘ComplexHeatmap’ (Gu et al., 2016) packages. ProBatch (Cuklina et al., 2021) was used to conduct quality control check for batch effects and peptide normality distributions.

## RESULTS

In this study, we identified 1047 proteins (unique accession numbers) in the gill tissue of mussels from the four acclimation groups and following heat stress. Of these, the number of significant proteins varied by acclimation conditions, with the greatest number of differentially abundant proteins in the low temperature acclimation groups, with 380 and 210 proteins among the low temperature–low food (LTLF) and low temperature–high food (LTHF) mussels, respectively. In the high temperature acclimation groups, there were 171 significant proteins in the high temperature–low food (HTLF) and 152 in the high temperature–high food (HTHF) mussels. These results support previous studies that show that acclimation to high temperatures and food ration confer higher tolerance to acute heat shock, whereas mussels without thermal conditioning and that are poorly fed display a lower thermal tolerance (Fitzgerald-Dehoog et al., 2012; Schneider et al., 2010). The differentially abundant proteins are involved in energy metabolism, oxidative stress responses, proteostasis and protein quality control, gene expression and translation, cellular structure and communication, and immune responses. Given the breadth of information collected in this study, this paper focuses on the proteomic response to heat shock with respect to core energy metabolism, one-carbon metabolism, oxidative stress, DNA chromatin modifications and DNA repair. As described above, the remaining information will be detailed in subsequent papers.

### Core energy metabolism

**Fig. 2.**
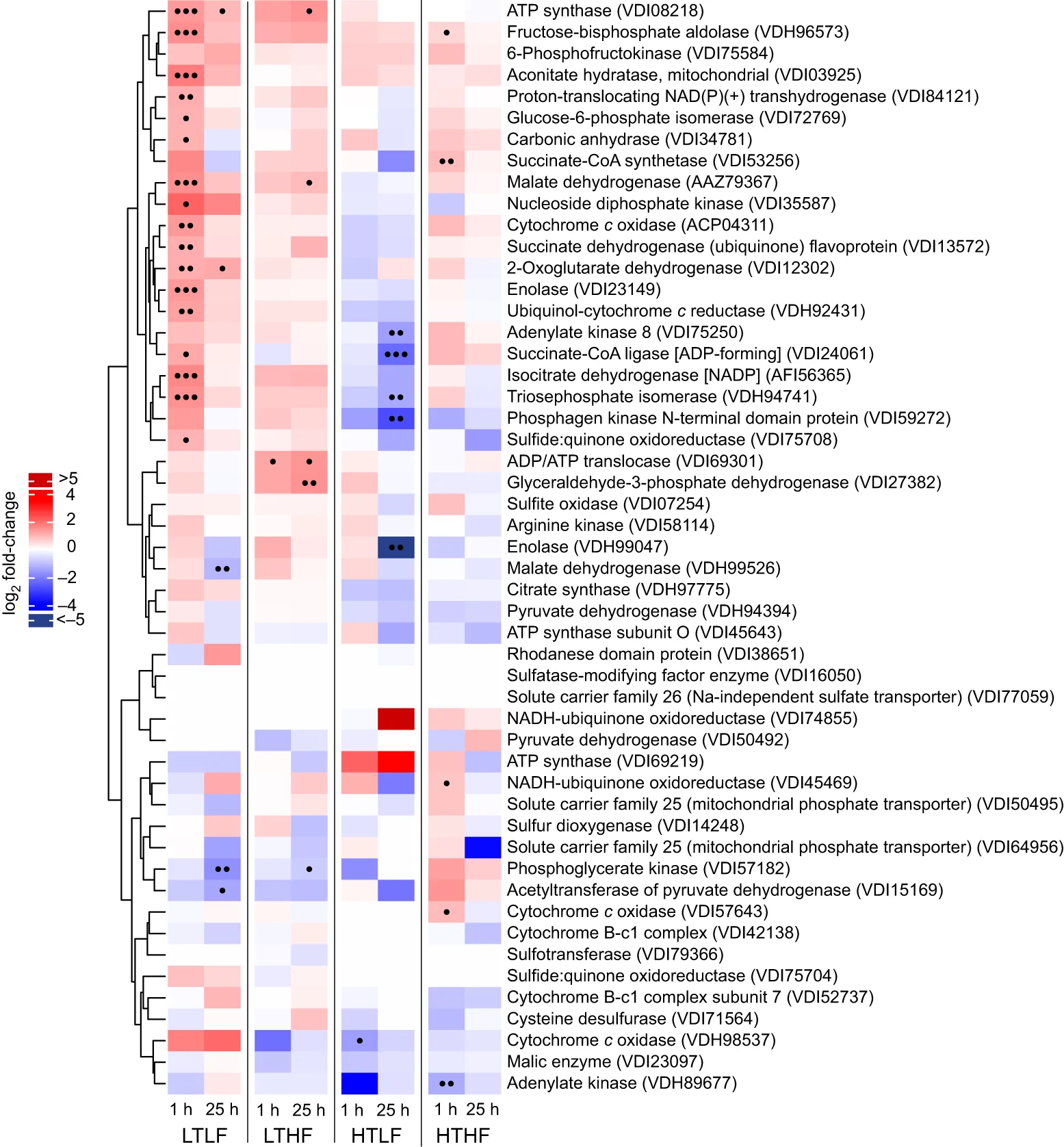
**Heatmap of core carbohydrate and sulfide metabolism proteins.** The heatmap shows log_2_ fold change in protein abundance from 1 or 25 h recovery relative to the baseline in each of the acclimation groups: low temperature–low food (LTLF), low temperature–high food (LTHF), high temperature–low food (HTLF) and high temperature–high food (HTHF). Increases in abundance are indicated by warm colors (reds), while decreases are cool (blues). Significance levels are shown by asterisks (***P<0.001, **P<0.01, *P<0.05). Proteins were identified using GenBank accession numbers.

Fifty-one proteins were identified within core energy metabolism, and 30 of these changed in abundance across acclimation groups and time points (Fig. 2). Most of the variation was observed in the LTLF mussels at 1 h, which increased abundance of many proteins involved in energy metabolism, suggesting a significant energetic demand following acute heat shock. In this group, two of the three glycolytic enzymes identified as part of the investment phase, i.e. fructose-bisphosphate aldolase and triose phosphate isomerase, and one of two enzymes identified as part of the pay-off phase, i.e. enolase, increased. Likewise, LTLF mussels increased most of the seven tricarboxylic acid (TCA) cycle enzymes (or their subunits) in response to heat shock. Nucleoside diphosphate kinase, converting GTP produced by the succinyl CoA synthetase to ATP (Michal and Schomburg, 2012), also increased. Further, we observed significant changes within one subunit for succinate dehydrogenase (SDH), cytochrome *c* oxidase (COX) and ATP synthase, representing complexes II, IV and V, respectively, and cytochrome *c*. The rapid increase in many enzymes involved in cellular respiration in LTLF gill tissues during exposure to heat shock suggest that these mussels are disproportionately impacted by heat shock compared with other acclimation groups.

In addition to the production of NADH as an electron carrier for ATP generation, core metabolism also contributes to the production of NADPH as a reducing equivalent for antioxidative reactions, such as those of the glutathione reductase-peroxidase and thioredoxin-peroxiredoxins reactions (Murphy, 2009; Tomanek, 2015). A proton-translocating transhydrogenase and NADP-dependent isocitrate dehydrogenase (NADP-IDH), which both increased in LTLF mussels, generate NADPH through distinct pathways. The former reduces NADP^+^ directly, whereas the latter oxidizes citrate to produce NADPH. Both help maintain a high ratio of reduced to oxidized glutathione (GSH/GSSG) to support antioxidative reactions and thus help maintain the cellular redox balance during oxidative stress (Sies and Jones, 2020). These changes raise the question of how reducing equivalents produced by the elevated TCA cycle enzymes in LTLF mussels are allocated – for ATP production (via oxidation by ETC) or for redox balance [via NADPH by NAD(P)H-transhydrogenase]. The subsequent return of NAD(P)H-transhydrogenase to base levels at 25 h in LTLF mussels suggests that the need for increased reducing equivalents for replenishing GSH, and thereby antioxidative capacity, was temporary.

**Fig. 3.**
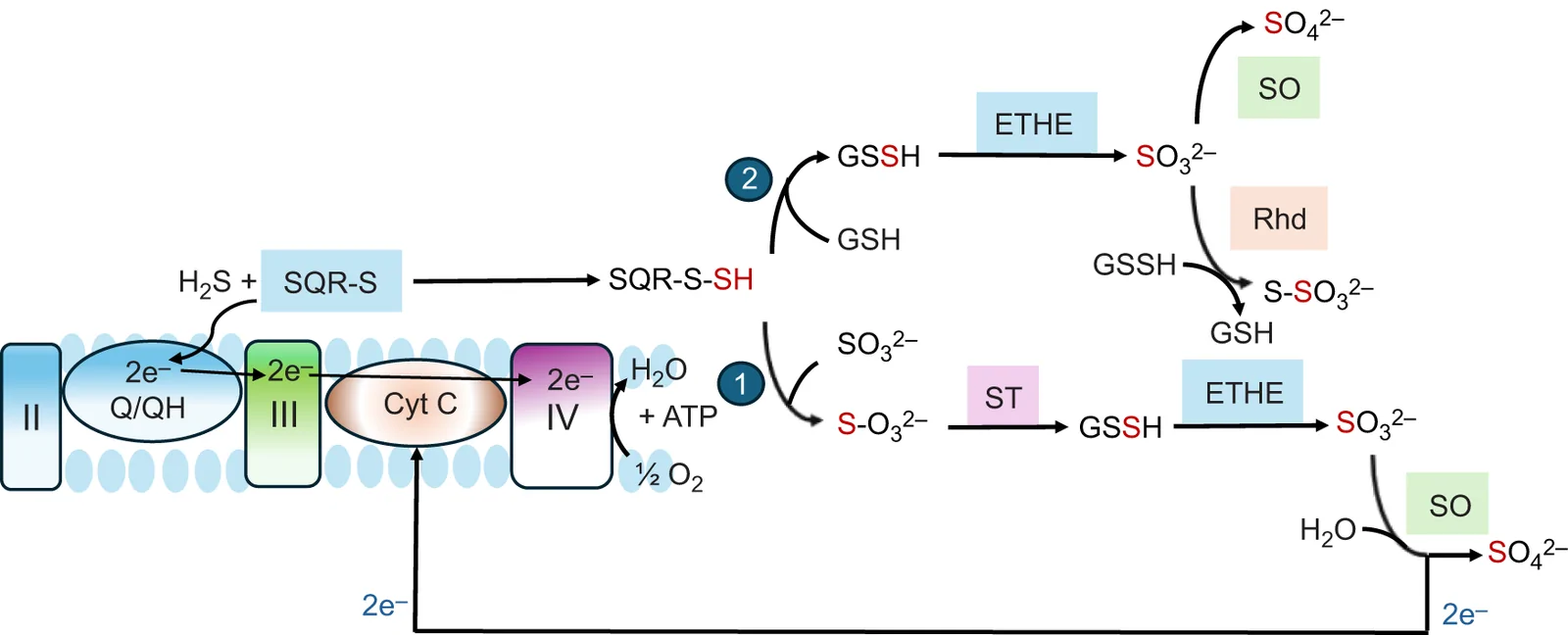
**Sulfide oxidation pathway.** Hydrogen sulfide detoxification and catabolism. H_2_S binds to sulfur quinone oxidoreductase (SQR) to form a persulfide, thereby delivering two electrons to the ETC (quinone) and fueling oxidative phosphorylation. In path 1, the persulfide is converted into thiosulfate by sulfurtransferase (ST) to glutathione persulfide (GSSH). Sulfide dioxygenase (ETHE) oxidizes GSSH to sulfite (SO_3_^2−^), which can be oxidized further by sulfur oxygenase (SO) to sulfate releasing additional two electrons to cytochrome *c*. In path 2, glutathione (GSH) is the initial mobile carrier and rhodanase (Rhd) forms thiosulfate (S_2_ SO_3_^2−^). Modified after Olson and Straub (2016).

The increase of sulfide:quinone oxidoreductase (SQR), forming a persulfide (SQR-S-SH), in LTLF mussels suggests that hydrogen sulfide (H_2_S) is used as an electron donor to the ETC (via quinone) and ultimately for ATP synthesis via oxidative phosphorylation during heat stress (Figs 2 and 3). The persulfide can combine with glutathione (GSH) to form glutathione-persulfide (GS-SH) through sulfur dioxygenase (ETHE), and can be further oxidized to sulfate by sulfur oxidase, producing additional electrons that can enter the ETC at cytochrome *c* (Olson and Straub, 2016). Given the role of GSH in sulfide oxidation, it is therefore suggestive to link the increase in SQR with the parallel increase in NADPH-generating reactions and the transsulfuration pathway producing glutathione (see below).

In comparison, abundance changes from mussels under other food–temperature combinations were limited, and often opposite of the response of the LTLF mussels. Although minimal, the responses within mussels from the high temperature acclimation groups suggest a robust energetic recovery. In HTLF mussels, we observed a decrease in phosphagen (adenylate kinase and phosphagen kinase), glycolytic (TPI and enolase), TCA cycle (succinate CoA-ligase) and ETC (cytochrome oxidase subunit 5a) enzymes, at either 1 or 25 h. Additionally, another isoform of adenylate kinase decreased in HTHF mussels, further supporting the conjecture that thermal conditioning enhances energy efficiency during stress (Buckley et al., 2001). The abundance changes of NAD(P)H-transhydrogenase in the HTHF mussels, although not significant, was similar to that of the LTLF group and reversed in HTLF mussels, possibly because HT-acclimated mussels are able to maintain a greater but not sufficient antioxidative capacity during acute heat stress. Very few proteins changed in the LTHF mussels, but an increase in ADP/ATP translocase in this group is noteworthy, suggesting that energetic demands are met via various means (e.g. carbohydrate metabolism versus P/ADP/ATP transport), depending on thermal and feeding histories. Thus, we propose that distinct ‘limiting factors’ of ATP production among acclimation groups necessitate different protein levels in response to acute heat stress.

We identified two fatty-acid binding proteins, all enzymes of mitochondrial fatty acid β-oxidation, two putative members of peroxisomal β-oxidation, and three enzymes involved in the synthesis of long-chain fatty acids (Fig. S1A). Several enzymes of mitochondrial fatty acid β-oxidation decreased at 1 and 25 h, including acetyl-CoA acetyltransferase in LTLF, and electron transfer flavoprotein and enoyl-CoA hydratase at 25 h in HTLF and HTHF mussels. Together, these results suggest that β-oxidation was downregulated, at least in mussels from HT acclimations during heat shock. Similarly, we identified several enzymes of the branched-chain amino acid (BCAA, or isoleucine, valine and leucine) pathways (e.g. four for valine catabolism), but only two changed. The E1 subunit of the α-ketoacid dehydrogenase complex (involved in the catabolism of all three BCAA) increased in HTLF mussels (Fig. S1), consistent with an increase of BCAA in heat-hardened mussels (Georgoulis et al., 2023). The oxidation of leucine and isoleucine may increase the efficiency of ATP synthesis and thereby mitigate the production of reactive oxygen species (ROS) (Wu et al., 2022). In contrast, 3-hydroxyisobutyrate dehydrogenase, involved in valine catabolism to succinyl-CoA only (Salway, 2017), decreased in HTLF mussels. This suggests that abundance changes in fatty-acid and BCAA metabolism are limited, mirroring the reduction of core carbohydrate metabolism in HT-acclimated mussels, indicating reduced energetic demands compared with LTLF mussels.

LTLF mussels heavily relied on core carbohydrate metabolism to produce reducing equivalents through core carbohydrate and sulfide metabolism for ATP synthesis and/or redox balance. The magnitude of the response within energy metabolism within this acclimation group suggests that acute heat shock causes significant cellular damage or stress in mussels with low food availability and no thermal conditioning, reinforcing the link between food ration and thermal tolerances as previously reported in this species (Fitzgerald-Dehoog et al., 2012; Schneider et al., 2010).

### One-carbon metabolism

**Fig. 4.**
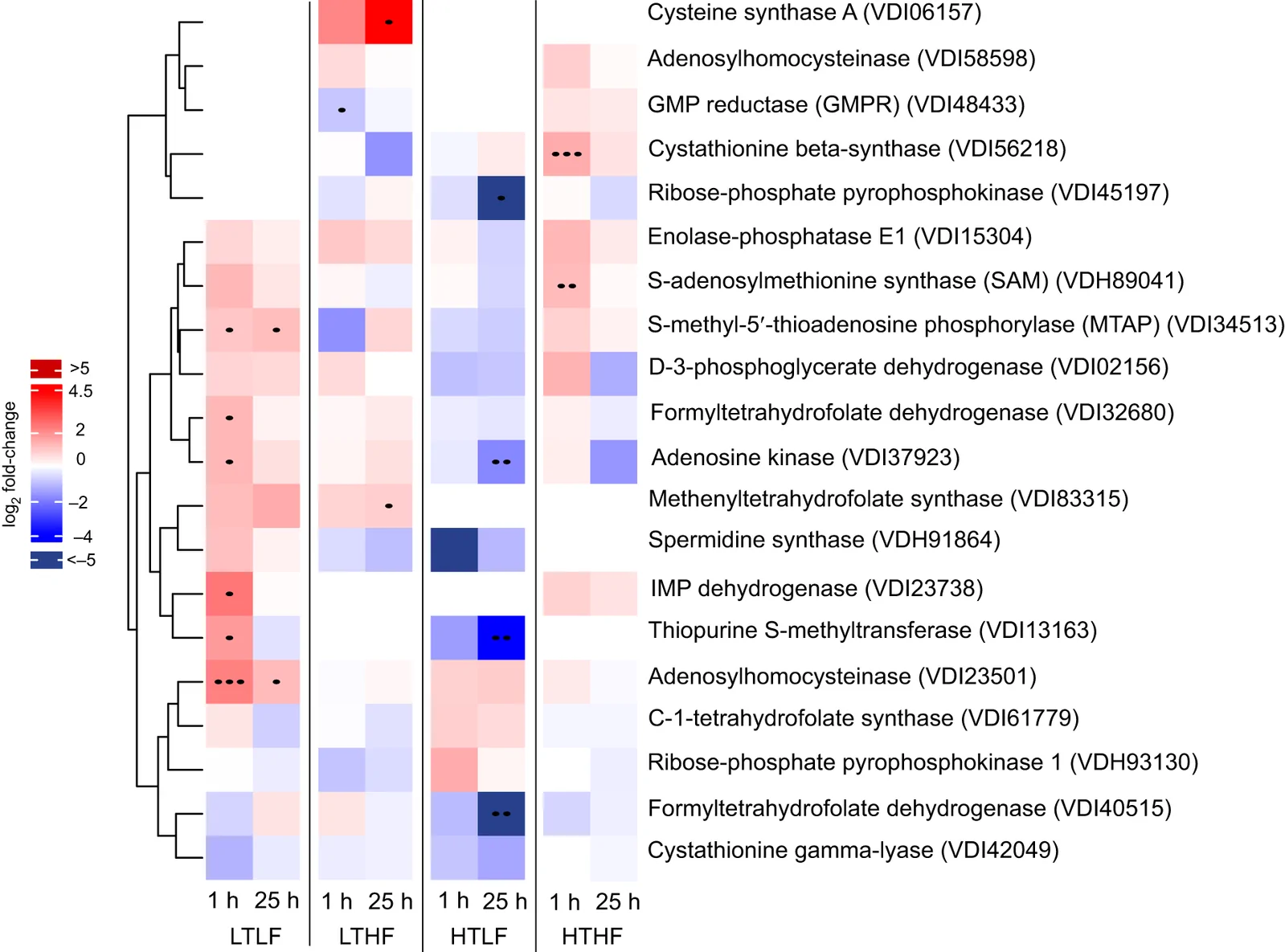
**Heat map of one-carbon metabolism proteins.** The heatmap shows log_2_ fold change in protein abundance from 1 or 25 h recovery relative to the baseline in each of the acclimation groups. For additional details, see Fig. 2.

**Fig. 5.**
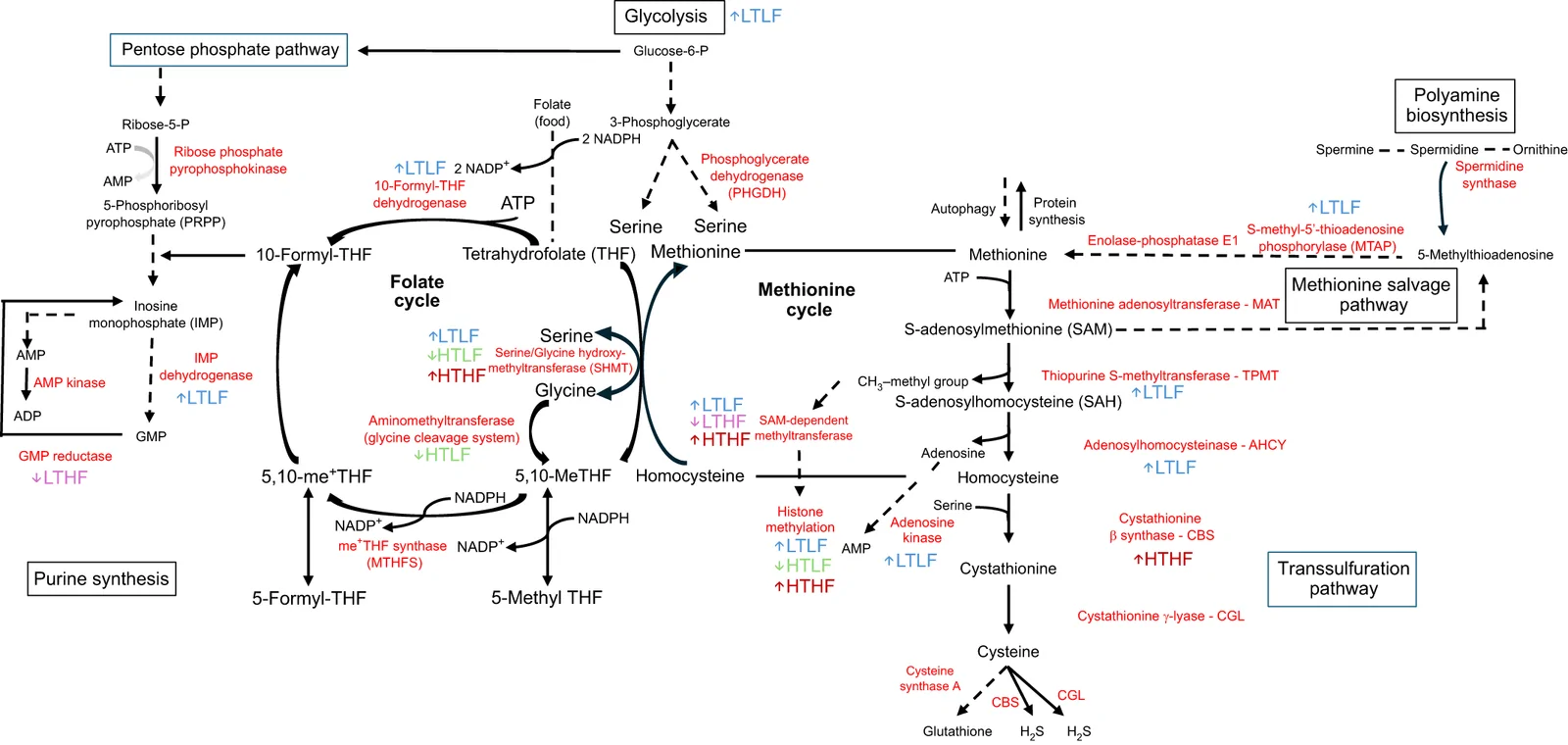
**Overview of one-carbon metabolism: the folate and methionine cycles and connecting pathways.** The folate cycle is coupled to the methionine cycle through the generation of methyl-tetrahydrofolate (THF), exchanging a carbon through the methylation of homocysteine to generate methionine. The folate cycle is characterized by the transformation of THF into products with one-carbon units that differ in oxidation states and support various biosynthetic pathways, including purine synthesis. The methionine cycle is receiving methionine and glycolytically derived serine to produce the substrate for histone/DNA/RNA methylation and simultaneously feeds into the transsulfuration pathway, which can produce cysteine, glutathione and hydrogen sulfide. The direction of the folate reactions is dependent on the NADPH/NADP^+^ ratio in the cytosol and mitochondria (based in part on Ducker and Rabinowitz, 2017; Locasale, 2013). Red: proteins identified. Changes in abundance are indicated as increases (up arrow) and decreases (down arrow) for different treatments. Solid black arrows indicate direct reactions, while dashed black arrows are multiple, indirect reactions between metabolites. Bi-directional arrows indicate reversible reactions.

Twenty proteins were identified as components of one-carbon metabolism (Fig. 4). One-carbon metabolism combines the independent but linked folate and methionine cycles to distribute methyl groups for the synthesis of proteins and nucleotides through amino acid homeostasis, thus serving as an integrator of nutrient status (Locasale, 2013). These pathways also help regulate epigenetic modifications through DNA/RNA and protein methylation as well as contribute to redox balance. The folate cycle is fed by folate acid (with biologically active forms reduced to tetrahydrofolate, THF), serine, glycine and methionine (Fig. 5), where it contributes methyl groups to purine (AMP/GMP) synthesis and affects NADPH/NADP^+^ balance. The direction of the cycle depends on the NADPH/NADP^+^ ratio in the cytosol and the mitochondria, where the folate cycle occurs (Ducker and Rabinowitz, 2017). Within the methionine cycle, S-adenosylmethionine (SAM) contributes methyl groups to SAM-dependent methylases, which serves as the universal methyl donor for histone, DNA and RNA-methyltransferases (Lu and Thompson, 2012), thus directly controlling chromatin and epigenetic modifications. This pathway may also increase the rate at which SAM contributes methyl groups to several additional methyltransferases (Fig. 5) and adenosine, serving as an activation signal for the mitogen-activated protein kinase (MAPK) pathway, and consequently AMPK-mediated upregulation of carbohydrate metabolism (Hardie et al., 2012). SAM is catabolized into homocysteine, which contributes to cysteine synthesis and, ultimately, the non-enzymatic antioxidant GSH, one of the most concentrated cellular metabolites, through the transsulfuration pathway (Sbodio et al., 2019). Our results suggest that the metabolic intermediates and products of the folate, methionine and connecting pathways are integral but often understudied regulators of the CSR.

As with core metabolism, one-carbon metabolism generally increased in LTLF mussels, whereas other acclimation groups had varying responses with respect to abundance changes within these pathways (Fig. 4). The increased abundance of methionine salvage (i.e. S-methyl-5′-thioadensoine phosphorylase), methionine cycle (i.e. thiopurine-S-methyltransferase and adenosylhomocysteinase) and transsulfuration pathway proteins likely contribute to increased homocysteine in mussels from the LTLF groups. Increases in phosphoglycerate dehydrogenase (PHGDH), along with parallel increases in serine/glycine hydroxy-methyltransferase (SHMT/GHMT) suggest concurrent activation of the folate cycle (Fig. 4) and THF production. The subsequent conversion to 10-formyl-THF via an increase in 10-formyl-THF dehydrogenase would provide precursors for purine synthesis through IMP dehydrogenase (Buey et al., 2022; Kozhevnikova et al., 2012). However, the state of both cycles depends on nutrient, metabolite and ATP production from core carbohydrate metabolism (Serefidou et al., 2019).

Within the other acclimation groups, few proteins changed abundance in these pathways (Fig. 4), e.g. SHMT linking the two cycles in HTHF mussels. The utilization of the folate and methionine cycles in these mussels may signify a primed response to rapidly and efficiently make cellular adjustments immediately following heat shock, as indicated by the response of core metabolic proteins in this group. Many of these proteins did not change abundance in the LTHF and HTLF mussels, again highlighting the importance of acclimation conditions on the metabolic support for the CSR and subsequent repair functions.

### Antioxidative proteins

During heat shock, oxidative eustress and distress can be affected by (1) scavenging of reactive oxidants (mainly reactive oxygen species, ROS), (2) protecting protein cysteines from oxidation, (3) repairing ROS-induced damage, (4) chelating ferrous iron (and other transition metals) to prevent the Fenton reaction, and (5) producing hydrogen peroxide for signaling (Sies and Jones, 2020). Oxidative stress can also be controlled by NADPH production to support ROS scavenging. Consistent with proteins within core and one-carbon metabolism, LTLF mussels increased numerous oxidative stress-related proteins identified in this study, further suggesting an initial redox imbalance.

**Fig. 6.**
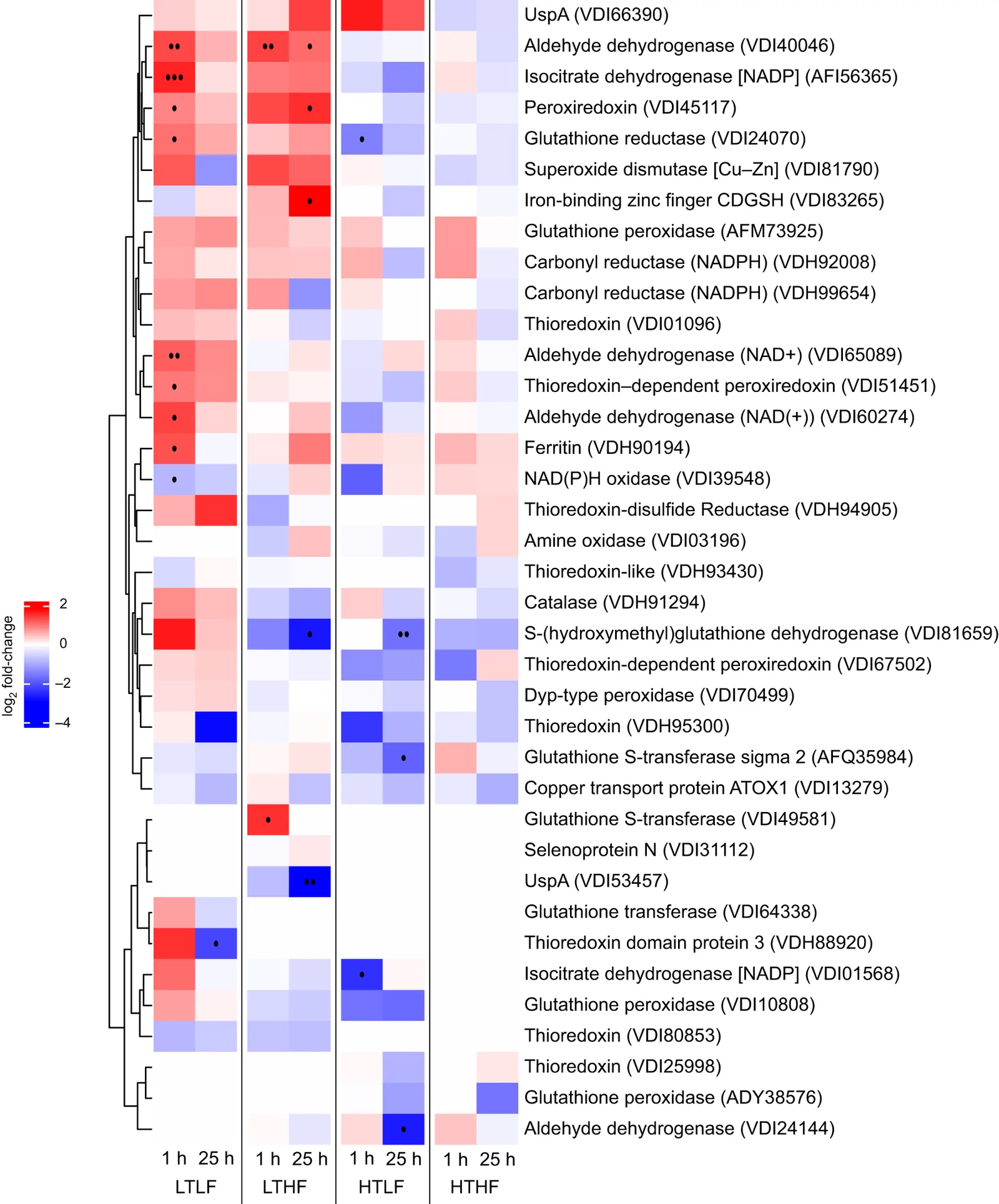
**Heat map of antioxidative proteins.** The heatmap shows log_2_ fold change in protein abundance from 1 or 25 h recovery relative to the baseline in each of the acclimation groups. For additional details, see Fig. 2.

Superoxide dismutases (SODs) are often the first step in scavenging ROS, by converting O_2_^−·^ to H_2_O_2_. Hydrogen peroxide is then converted to water by either catalase, glutathione peroxidase or peroxiredoxins. In this study, Cu-Zn SOD did not change significantly in our treatment groups (not shown), consistent with our previous study (May and Tomanek, 2024). This is possibly because Cu-Zn SOD can undergo post-translational modifications in response to heat shock rather than changes in abundance, as indicated by gel-based changes (Vasquez et al., 2017, 2020). Although there were no observed changes in SODs, LTLF mussels (Fig. 6) increased two cytosolic peroxiredoxins (PRX6 and another cytosolic PRX isoform), indicating a need for increased hydrogen peroxide scavenging (Rhee and Kil, 2017). This would prevent H_2_O_2_ from reacting with ferrous iron via the Fenton reaction (Fujii et al., 2022; Tomanek, 2015). Together, with decreased thioredoxin (TRX)-domain protein isoforms and increased thioredoxin-disulfide reductase, it appears that heat shock in LTLF mussels causes high turnover of TRX and perhaps overwhelms the PRX-TRX antioxidant system. We also observed increased ferritin, the main Fe^2+^-binding protein, in LTLF mussels, again supporting the need to sequester ferrous iron to prevent the Fenton reaction.

The targeted antioxidant response in LTLF mussels is further supported by changes in the glutathione redox system and aldehyde detoxification. The increased glutathione reductase (GR) coupled with one-carbon metabolism results (i.e. transsulfuration) suggests an active need to maintain GSH. Further, glutathione can help protect protein thiols from oxidative damage via glutathione S-transferase (GST), which may also exert peroxidase activity and participate in the synthesis of the lipid mediator prostaglandin (Sheehan et al., 2001). The abundance of two GST isoforms changed significantly (increasing in LTHF and decreasing in HTLF mussels), indicating an acclimation-dependent need to maintain protein thiol groups during the CSR. Reactive aldehydes (e.g. 4-HNE, 4-oxononenal and malondialdehyde) formed by oxidative-stress-induced lipid peroxidation can also interact with thiols to form Michael adducts. These aldehydes are detoxified by carbonyl reductase and aldehyde dehydrogenase (ALDH) (An et al., 2024); the latter also converts protein carbonyl groups into their corresponding alcohols, requiring NADPH. Although carbonyl reductase did not change, three ALDH isoforms increased in LTLF mussels (Fig. 6). The ALDH family of enzymes participate in several metabolic pathways (Singh et al., 2013), including the oxidation of branched-chained amino acids and the synthesis of prostaglandins. Maintenance of high NADPH levels support a robust increase in antioxidant system proteins following heat shock, suggesting a strong link to the folate cycle, NADP-IDH, the pentose–phosphate pathway and several other reactions (Tomanek, 2015), as described above for LTLF-acclimated mussels.

### Chromatin remodeling, transcriptional regulation and DNA repair

**Fig. 7.**
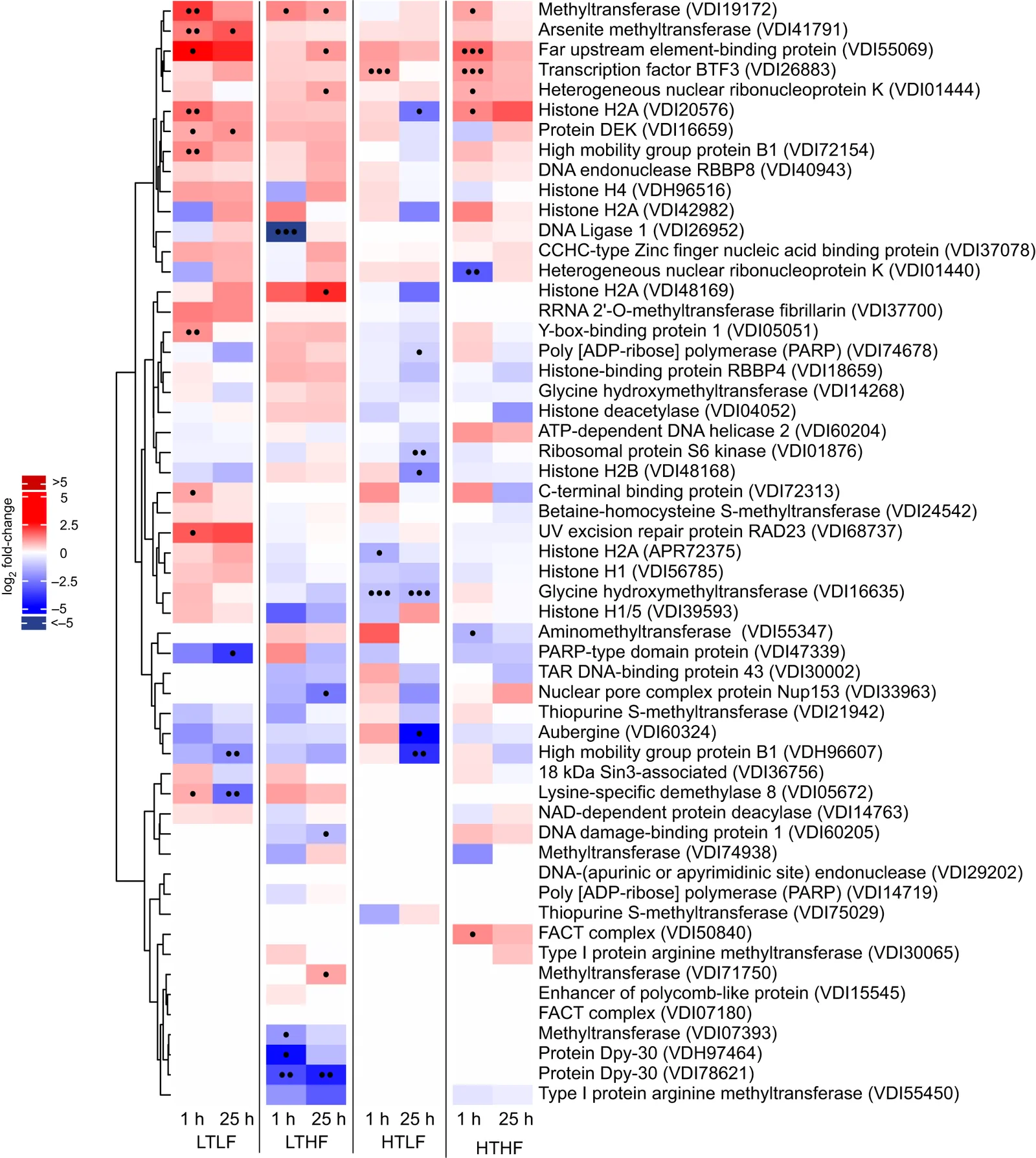
**Heat map of chromatin structure, transcription and DNA repair proteins.** The heatmap shows log_2_ fold change in protein abundance from 1 or 25 h recovery relative to the baseline in each of the acclimation groups. For additional details, see Fig. 2.

Acute stress leads to the immediate rearrangement of regions of the genome that are necessary to respond to stress. This includes formation of chromosomal puffs during the heat shock response to prepare for transcription of stress-induced proteins (Ritossa, 1962), as well as repressing other regions to reestablish cellular homeostasis in response to environmental change. Transcriptional control is regulated by modifications of histone proteins via histone post-translational modifications [e.g. phosphorylation, acetylation, methylation and poly (ADP)-ribosylation]. Several transcriptional co-activators and co-repressors were identified and changed in a food-by-temperature-dependent fashion (Fig. 7). Within the LTLF mussels, there were many histone and associated proteins that increased in abundance, providing evidence that mussels altered chromatin structure and transcriptional control in response to heat shock (Venkatesh and Workman, 2015). We observed acclimation-specific responses in H2A, a histone isoform associated with transcriptional repression and recruitment of DNA repair proteins, similar to a study showing an effect of diet on H2B in *C. elegans* during starvation (Zhu et al., 2023). Additionally, we found that numerous proteins involved in PTMs of histones increased in abundance in the LTLF group, including high mobility group (HMG) proteins and binding protein DEK. Collectively, these regulate histone acetylation (Ishida et al., 2020). There were also several methyltransferases, including three SET-domain methyltransferase modifying histones (Dillon et al., 2005; Herz et al., 2013), and an arsenite methyltransferase that increased in LTLF mussels. The changes in SET-domain methyltransferases represent a likely connection with the SAM produced by the methionine (i.e. the TPMT reaction) and folate cycles (e.g. SHMT; Fig. 5). This is in congruence with a study on eastern oyster gill, where GO enrichment analysis of the methylated genome showed SAM-dependent methyltransferases and a proton-coupled folate transporter differentially methylated in populations inhabiting different salinities (Johnson and Kelly, 2020).

Changes to other proteins involved in histone modification also correspond to other patterns observed in LTLF mussels. For example, we observed increased lysine-specific demethylase 8 (KDM8), which cleaves the N-terminal tail of histone H3 at mono-methylated lysine residues, thus regulating the expression of antioxidant genes and proteins (e.g. catalase and SOD) and activating DNA repair pathways (Separovich and Wilkins, 2021). The NAD-dependent C-terminal binding protein (CtBP) also increased, helping respond to DNA damage and recruit histone-modifying enzymes to enhance transcriptional repression via acetylation/deacetylation (Chinnadurai, 2002). Poly (ADP-ribose) polymerase (PARP) decreased; this protein is normally activated by DNA strand breaks and determines whether cells are being repaired or die following stress by weakening histone–DNA interactions (Luo and Kraus, 2012). In this context, it is useful to consider that competitors for the NAD^+^ required by PARPs are calorie- and NAD-dependent deacylases (i.e. sirtuins), which are mostly involved in gene inactivation. Although we did not observe sirtuin increases in this study, we have shown previously that the mitochondrial sirtuin 5 (Sirt5) displays a species-specific role during heat stress in *Mytilus* (Vasquez et al., 2017) and that sirtuin activity is altered in mussels acclimated to varying food rations (May and Tomanek, 2024). Overall, LTLF mussels respond to heat stress with increased abundances of proteins in chromatin remodeling through histone modifications, activating transcription globally (e.g. HMG proteins), but also via selective transcriptional control (i.e. CtB, DEK and PARP).

Several proteins identified are likely involved in DNA damage repair given their chromatin-modifying function (Hakem, 2008). In addition to CtBP and DEK, nine proteins were identified that are involved in DNA damage recognition and repair, with two (e.g. RAD23 and YB-1) that increased in LTLF only (Fig. 7). DNA ligase 1 and DNA-(apurinic or apyrimidinic site) endonuclease (APE1) play important roles in the base excision repair (BER) pathway (Hegde et al., 2008; Sattler et al., 2003), whereas DNA damage-binding protein 1 (DDB1) and UV excision repair protein RAD23 are key components of the nucleotide excision repair (NER). Although also playing a role in stabilizing mRNAs, Y-box-binding protein 1 (YB-1) binds to DNA and interacts with PARP1, enhancing DNA repair (Naumenko et al., 2022), suggesting that LTLF mussels experienced DNA damage requiring upregulation of BER and NER pathways (Hakem, 2008).

Responses within the other acclimation groups also warrant discussion. For all mussels acclimated to low temperature, we observed changes in histone methylation, indicating that regardless of food, continuous aerial exposure leads to widespread chromatin modifications. For mussels acclimated to high temperature (i.e. HTLF), core histones are being modified and decreased, likely increasing transcription. This illustrates that HT acclimation buffers the initial challenges of a more severe heat stress but also requires an additional transcriptional response during recovery. Furthermore, transcription factor BTF3 binds to TATA and CAAT box sequences, forming a stable complex with RNA polymerase II – which increased in HT-acclimated mussels – and is implicated in regulating DNA damage repair (Zhang et al., 2021). Although single- and double-stranded DNA breaks in response to heat stress have been observed in *Mytilus* hemocytes before (Yao and Somero, 2012), their dependence on food-by-temperature acclimation is novel. HTHF mussels increased histones and methyltransferases, and possibly activated Nrf2, an important transcription factor of antioxidants, via the FACT complex, suggesting a more targeted response than was observed in the other groups. The patterns of histone changes provide an acclimation-specific response to heat stress, indicative of the role of epigenetic modifications in initiating food-related longer-term responses to changes in temperature.

## DISCUSSION

**Fig. 8.**
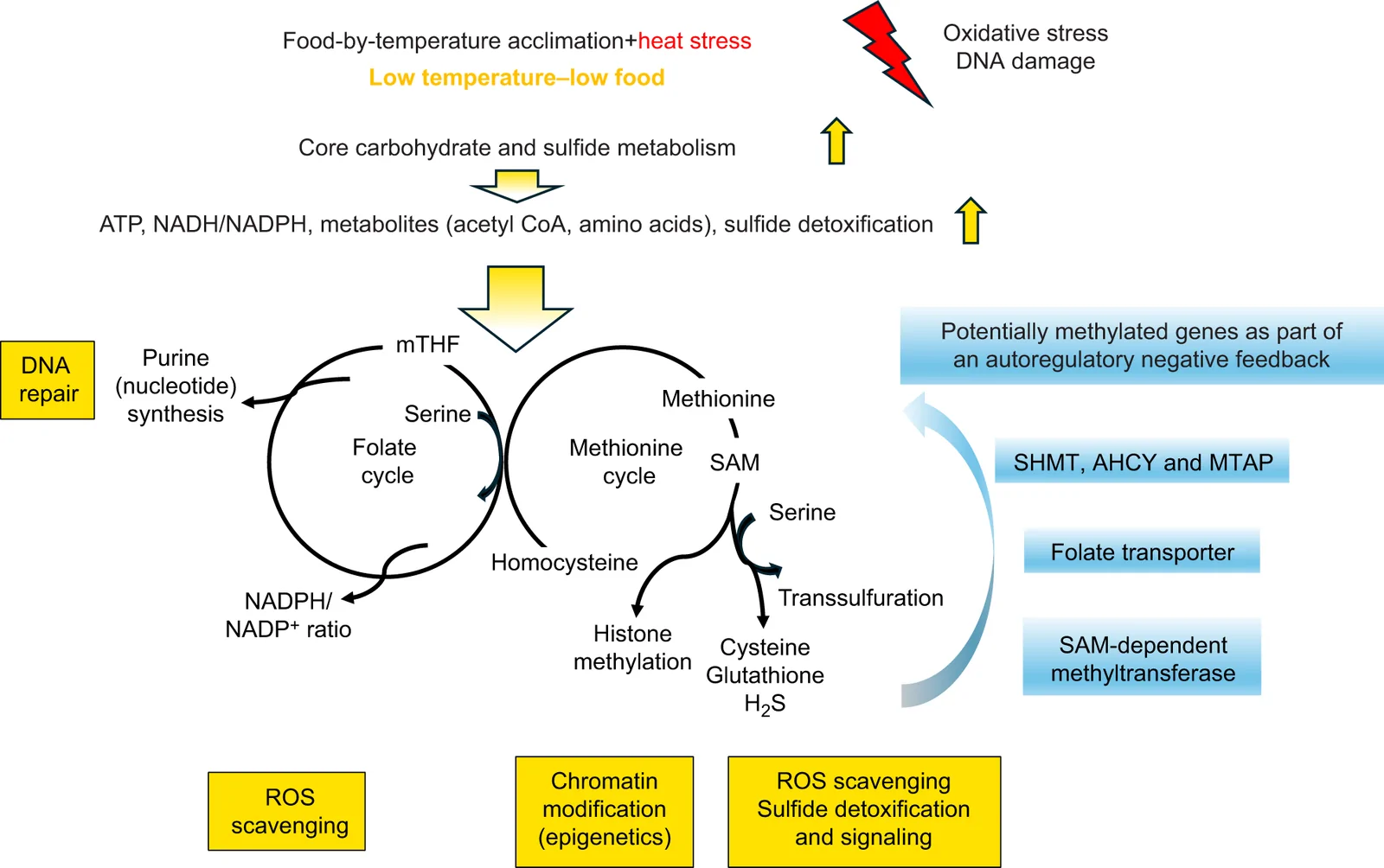
**Conceptual diagram showing the acute heat shock response in marine mussels under food limitation.** In addition to the damage experienced by heat stress and the simultaneous upregulation of metabolic pathways, we hypothesize one-carbon metabolism playing a role as an autoregulatory feedback linking metabolism and epigenetic modifications through the methylation of genes related to the fluxes of the pathways contributing to it, i.e. folate and methionine cycle.

In this study, we found that the response to an acute heat stress at the tissue level is dependent on food-by-temperature acclimation conditions. In contrast to the three other conditions, LTLF-acclimated mussels showed an upregulation of ROS scavengers and DNA repair proteins, indicating that they experienced greater cellular stress. Simultaneously, they increased proteins of core carbohydrate and sulfide metabolism, indicating a need to increase NADH, NADPH, ATP and metabolite production. Specifically, they increased the abundance of many enzymes in one-carbon metabolic and connecting pathways, which were likely fed by increased metabolite production from core metabolism (Fig. 8). Our results suggest that the role of the folate cycle may represent the main source of NADPH, in addition to the NADP-isocitrate dehydrogenase reaction, to provide H_2_O_2_ scavengers such as glutathione peroxidase and peroxiredoxin with reducing power. It also may produce precursors for purine synthesis to mitigate DNA damage. Furthermore, the methionine cycle likely plays a key role in the response of LTLF mussels, given the increases in several of its enzymes, with S-adenosylmethionine (SAM) being the main metabolite on which histone methyltransferases depend on, linking metabolism to chromatin modifications, as has been documented for other cellular systems (Katada et al., 2012; Lu and Thompson, 2012; Turner, 2009). The simultaneous increased levels of enzymes of the folate and methionine cycle and several SAM-dependent histone methyltransferases not only helps repair heat-induced DNA damage to maintain genome integrity, but may also lay down epigenetic markers for primed transcriptional responses to subsequent exposures to heat shock, even on a short or cyclical time frame. As such, we hypothesize that the lack of a global response in other mussels likely occurred at the onset of acclimation and was not captured at the time points sampled. Thus, mussels from the LTLF acclimation were less equipped than other treatment groups to respond to acute heat stress.

### Importance of one-carbon metabolism in the heat shock response

The proposed food-by-temperature dependence of the CSR between one-carbon metabolism (especially the methionine cycle) and chromatin structure raises several questions on how such environmental variation affects the epigenome of marine bivalves. One is how closely linked the SAM-dependent activities of (SET domain) histone methyltransferases are linked to DNA methylation and thus to epigenetic modifications. Although they comprise different families of methyltransferases, chromatin modifications regulate access to DNA methylation sites (Cedar and Bergman, 2009). But histone and DNA methylation both use SAM as the main methyl donor (Serefidou et al., 2019). Marine invertebrates have sparsely methylated genomes but can change DNA methylation in response to environmental change, e.g. warming (Marsh and Pasqualone, 2014). Although there is mixed evidence for how DNA methylation affects expression in marine invertebrates, a recent study on *M. californianus* showed that promoter methylation regulates expression altogether, whereas gene body methylation fine-tunes expression (Hiebert et al., 2025 preprint), likely regulating transcriptional noise (Venkataraman et al., 2024). However, specifics of which gene’s expression is controlled, and how, is rarely given in epigenetic studies (but see below). Metabolomic and proteomic studies have shown increases in methionine adenosyltransferase (MAT) and S-adenosylhomocysteine in the gills of *M. californianus* during high tide (Connor and Gracey, 2012; Elowe and Tomanek, 2021), as well as homocysteine after heat hardening in *M. galloprovincialis* (Georgoulis et al., 2023), suggesting a close link between the activity of the methionine cycle and the highly fluctuating tidal environment.

Overall, one-carbon metabolism emerges as a key hub to modify chromatin structure, via the methionine cycle, and contributes to the synthesis of NADPH and ROS scavengers, via the folate cycle, in response to aerial heat stress in LTLF mussels. Given that methionine cycle enzymes (i.e. MAT) and metabolites are known to affect histone and DNA methylation (Serefidou et al., 2019), together with the findings of changing metabolites, we can hypothesize that LTLF mussels may utilize these pathways to sense tidally fluctuating diet-based variation and thermal conditions similar to mammals (Mentch et al., 2015). In this context, it is interesting that LTLF mussels increased S-methyl-5′-thioadenosine phosphorylase (MTAP) of the methionine salvage pathway, recruiting 5-methylthioadenosine from polyamines (Fig. 5). Is this activity affecting epigenetic markers? Among the enzymes that were found to be differently methylated at their promoter region based on methionine restriction in human cells were several enzymes of one-carbon metabolism, including SHMT, AHCY and MTAP (Mentch et al., 2015). Additional evidence that environmental variation may modify the link between one-carbon metabolism and histone and DNA methylation comes from a survey of differently methylated genes from oysters along a salinity gradient, showing an enrichment for the SAM-dependent methyltransferase functional category and a folate transporter (Johnson and Kelly, 2020). We therefore propose that one-carbon metabolism represents an autoregulatory feedback mechanism of tidally fluctuating conditions, modified by food ration, that lays down short-term epigenetic markers to coordinate the CSR (Fig. 8).

### The role of sulfide in the food-dependent cellular stress response

Although it is plausible that LTLF mussels use sulfide oxidation for an additional pathway for ATP production, similar to the upregulation of its core metabolism, as has been observed under hypoxic conditions in the lugworm (Völkel and Grieshaber, 1997), the question is where the hydrogen sulfide is generated. Our data point to the transsulfuration pathway as a possible source. When homocysteine is converted to cystathionine by cystathionine β-synthase (CBS), using glycolytically derived serine, it produces cysteine (Sbodio et al., 2019). Although CBS can use homocysteine and cysteine, cystathionine γ-lyase (CGL) only requires cysteine to produce H_2_S (Fig. 5).

A key branching point is the partitioning of homocysteine towards either remethylation back to methionine (by the folate cycle) or transsulfuration. High SAM levels can act as an allosteric activator of CBS while simultaneously inhibiting the folate cycle, shifting homocysteine towards transsulfuration and H_2_S production (Sbodio et al., 2019). In contrast, higher SAM levels can inhibit methyltransferases and tilt the methionine cycle towards remethylation and transsulfuration. How this portioning is realized under our treatments is not clear from our data, but low food conditions may limit methionine, creating conditions that cause an upregulation of transsulfuration to maintain levels of cysteine but also increase H_2_S production (Hine et al., 2015; Mentch et al., 2015). Several simultaneous changes seem to contribute to reconfigure the fluxes along these pathways upon heat stress in LTLF mussels: the upregulation of the methionine salvage pathway, possibly augmented by polyamines, increases in SAM-dependent methyltransferases and changes in protein synthesis requiring methionine (Tomanek et al., 2026 preprint). We hypothesize that these changes aggregate to an increase in H_2_S, which not only serves as an antioxidant, but is also essential to obtain the beneficial effects of dietary restriction (Hine et al., 2015). Finally, H_2_S oxidized to thiosulfate by SQR can be further oxidized to sulfite or sulfate, which that can serve as final electron acceptors (Olson et al., 2013).

Furthermore, the H_2_S mimic Na_2_S can affect feeding rates as it reduces gill ciliary beat frequency, depending on levels of 5-hydroxytryptamine (serotonin), which stimulates lateral ciliary activity (Doeller et al., 1999). As ciliated cells make up 40% of the *Mytilus* gill mass and consume >90% of the oxygen consumed by the gill (Clemmensen and Jøgensen, 1987), a reduction in ciliary activity may provide LTLF mussels with additional energy during heat stress.

### Effects of food on the heat shock response

The effect of food on these pathways is an indication that strict control and reporting on feeding conditions is as necessary as reporting thermal conditions applied to an experimental design. Several of the changes we observed in LTLF mussels are consistent with previous studies. For example, heat-hardening followed by an acute increase in temperature in *M. galloprovincialis* has shown an increase in ETC activity, NADH dehydrogenase and COX expression, and aldolase activity in either mantle or posterior adductor muscle (Feidantsis et al., 2020; Georgoulis et al., 2021). Interestingly, the mussels in these studies were starved during the hardening period and then fed 0.5% dry mass g^−1^ mussel mass during the increase in temperature, more closely matching our low-food treatment. Proteomic studies using 2D-gel electrophoresis also showed similar results to ours in *Mytilus* congeners during heat stress in the gill (Tomanek and Zuzow, 2010; Vasquez et al., 2017), which would have lacked thermal conditioning and excess food. These studies suggest that our understanding of the heat shock response in marine bivalves may be limited to animals least poised to combat cellular stress. At the organismal level, numerous studies have shown that thermal conditioning or food can modulate the response to acute heat shock (Buckley et al., 2001; Fitzgerald-Dehoog et al., 2012; Moyen et al., 2020; Schneider et al., 2010). The muted response of mussels acclimated to warmer aerial temperatures and/or high food availability suggest that, in nature, mytilid mussels may be better primed for acute heat shock than laboratory-based studies suggest.

### Future directions

There are connections to these results with food-dependent responses of mRNA selection, splicing, ribosomal protein composition, the translation, chaperoning and degradation of proteins that we chose to report in a companion paper (Tomanek et al., 2026 preprint). The identification of three- to four-fold more proteins in our current proteomic workflow in comparison with previous 2D-gel analyses provides much deeper insights into the dynamics of the proteome and allows for the generation of several hypotheses to test in future studies. One goal of these studies is to connect the cellular-level changes with responses at the whole-organism level, and testing which potential regulatory mechanisms link different levels of organization. For example, although mussels acclimated to low food ration significantly reduced clearance rates with heat shock, they only did so when we applied sirtuin (sirtuins function as NAD-dependent deacylases) inhibitors and not in response to heat shock alone (May et al., 2021). Low food ration also increased sirtuin activity and was correlated with SOD activity (May and Tomanek, 2024). Furthermore, because sirtuins remove acyl groups such as acetyl and succinyl, post-translational modifications from proteins that are also metabolites of core carbohydrate metabolism, these results connect the increase in core carbohydrate metabolism of LTLF mussels to higher level performances, i.e. clearance rates, during heat shock.

Furthermore, it would be interesting to test whether the methionine cycle-dependent SAM-methyltransferases play a parallel role as sirtuins during heat stress in a food-dependent fashion. This could be achieved by inhibiting key enzymes of the cycle, e.g. methionine adenosyltransferase, and simultaneous whole-genome bisulfite sequencing. Such an approach could reveal a biochemical mechanism linking food ration to the cellular stress response and thermal tolerance.

## Supplementary Material

10.1242/jexbio.252105_sup1 Supplementary information
