# Supplementary material for: Food-dependent activation of one-carbon metabolism during heat shock in Mytilus californianus
Source: J Exp Biol. 2026 Aug 11;229(15):jeb252105. doi: 10.1242/jeb.252105 (PMC13535095; doi:10.1242/jeb.252105)
Supplement: Supplementary file 1 [file jexbio-229-252105-s1.pdf]

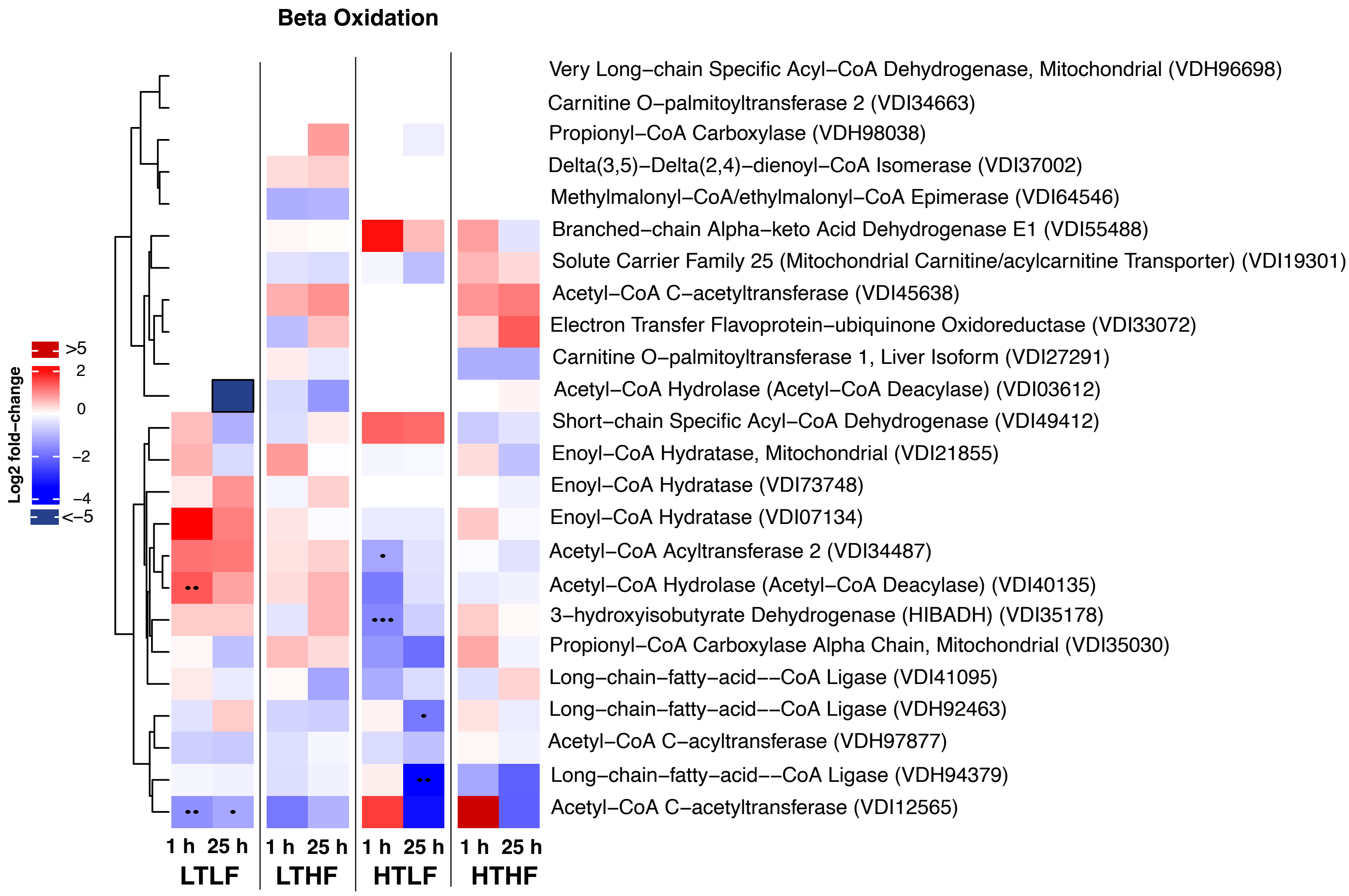

Fig. S1. Heatmap of fatty-acid (β-oxidation) metabolism. The heatmap shows $\log_2$ fold change in protein abundance from 1 h or 25 h recovery relative to the baseline in each of the acclimation groups: low temperature-low food (LTLF), low temperature-high food (LTHF), high temperature-low food (HTLF), and high temperature-high food (HTHF). Increases in abundance are indicated by warm colors (reds), while decreases are cool (blues). Significance levels are shown by dots, where ••• signifies $p < 0.001$, •• is $p < 0.01$, and • is $p < 0.05$. Proteins were identified using GenBank accession numbers.

Journal of Experimental Biology • Supplementary information
